# Impact of diabetes on the risk of bedsore in patients undergoing surgery: an updated quantitative analysis of cohort studies

**DOI:** 10.18632/oncotarget.14312

**Published:** 2016-12-27

**Authors:** Mining Liang, Qiongni Chen, Yang Zhang, Li He, Jianjian Wang, Yiwen Cai, Lezhi Li

**Affiliations:** ^1^ Department of Psychiatry, The Second Xiangya Hospital, Central South University, Changsha, Hunan Province, China; ^2^ Department of Nursing, The Second Xiangya Hospital, Central South University, Hunan Province, Changsha, China; ^3^ Nursing Teaching and Research Institute, Medical College of Guangxi University of Science and Technology, Liuzhou, Guangxi Province, China

**Keywords:** diabetes, bedsore, surgery, meta-analysis

## Abstract

Diabetes is a major cause of morbidity for patients undergoing surgery and can increase the incidence of some postoperative complications such as bedsores. We conducted a meta-analysis of observational studies to examine whether patients with diabetes undergoing surgery had high risk of bedsore. We performed a systematic literature search in Pubmed, Embase and the Cochrane Library Central Register of Controlled Trials database from inception to November 2016. Studies were selected if they reported estimates of the relative risk (RR) for bedsore risk in postoperative diabetic patients compared with that of in non-diabetic patients. Random-effects meta-analysis was conducted to pool the estimates. A total of 16 studies with 24,112 individuals were included in our meta-analysis. The pooled RR of bedsore development for patients with diatetes was 1.77 (95% CI 1.45 to 2.16). The results of subgroup analyses were consistent when stratified by surgery type, study design, research region, sample size, inclusion period, analysis method and study quality. There was evidence of publication bias among studies and a sensitivity analysis using the Duval and Tweedie “trim-and-fill” method did not significantly alter the pooled results (adjusted RR 1.17, 95% CI 1.02 to 1.36).This meta-analysis provides indications that diabetic patients undergoing surgery could have a higher risk of developing bedsores. Further large-scale prospective trials should be implemented to comfirm the association.

## INTRODUCTION

Bedsore, also known as pressure ulcer, is a common cause of prolonging length of hospital stay for patients with surgery. It has been reported that the length of hospital stay of surgical patients could increase by 3.5 to 5 days on average when a bedsore occurs [1, 2]. For some severe cases, the length of stay for bedsores could even be longer than 15 days [3], which adds tremendous financial burden on the patient and healthcare facility.

Several risk factors and aetiologies have been reported to contribute to the development of bedsores during perioperative period. Traditionally, it is considered that patients with advanced age, malnutrition (lower levels of hematocrit or albumin), poor circulation or smoking may have a higher risk of bedsores [4–7]. Moreover, for patients with surgery, some other factors such as anesthesia and surgery type, length of surgery, patient position during the surgery, warming or moisture devices used, and padding type the patients used [8–11] could also affect the development of bedsores.

Numerous studies have explored the role of patients with preexisting diabetes on the development of bedsore. Despite the fact that some studies have reported significant association between diabetes and risk of surgery-related bedsore, some others have reported varying results on this association. It was noted in several studies that surgical patients with diabetes had higher risk of bedsore than those without diabetes [12–18], while still others showed null association [19–23]. Although two previous meta-analyses have explored this topic and found significant association between diabetes and surgery-related bedsore [24, 25], limited sample size and significant heterogeneity which was not sufficiently examined made the results less reliable. Therefore, there is an urgent need to update the evidence of association between preexisting diabetes and surgery-related bedsore.

## RESULTS

### Search and selection of studies

**Figure 1:**
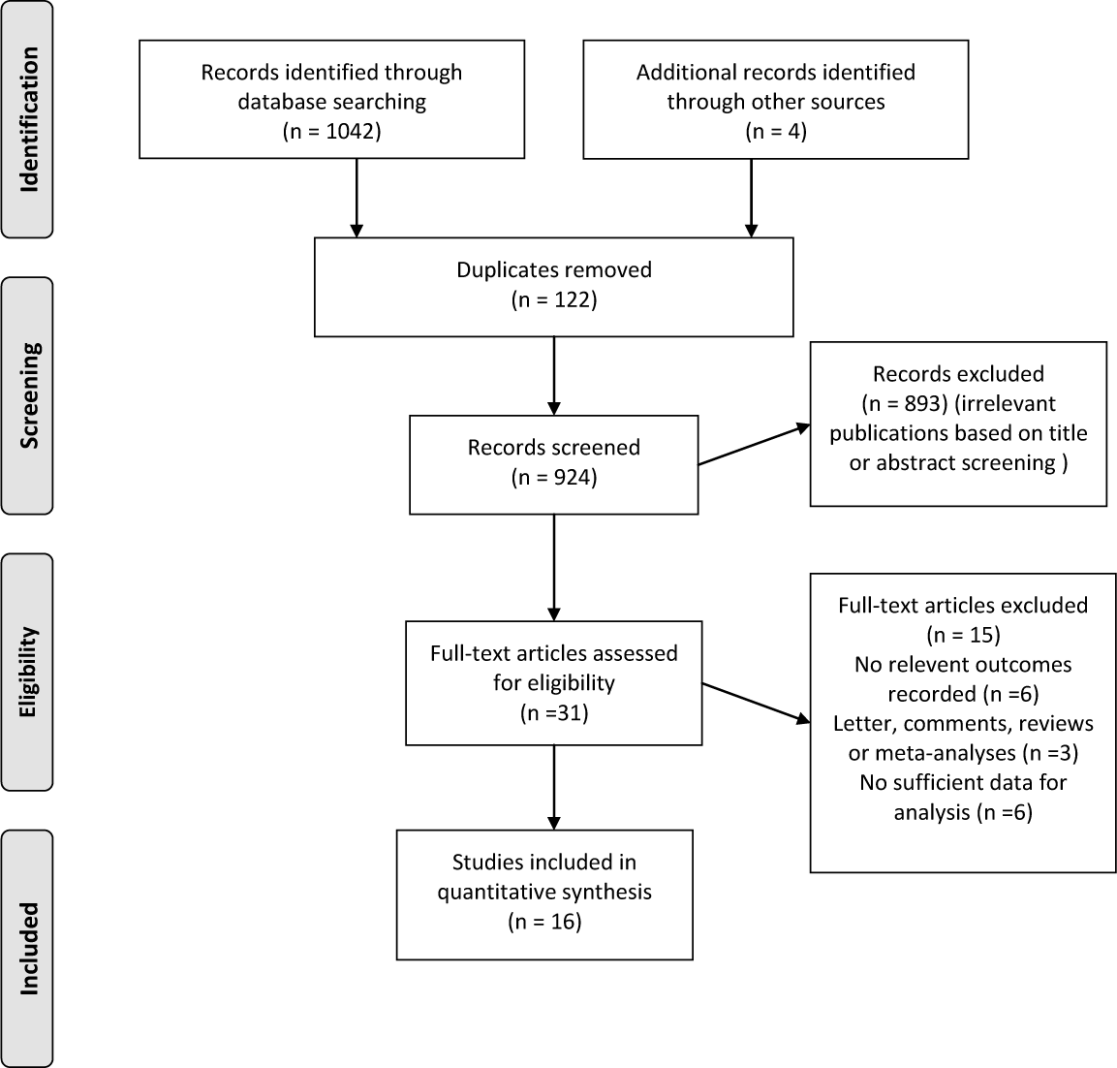
Flow diagram of the study selection

The initial literature search yielded 1046 abstracts of which 31 were considered potentially relevant for full-text review. Totally, 16 studies including 24,112 participants met our eligibility criteria and were involved in the meta-analysis [12–23, 26–29]. Figure 1 gives the detailed process for study selection of this meta-analysis.

### Study characteristics

Table 1 presents the baseline characteristics of the 16 included studies. In summary, the included studies were published between 1994 and 2013 with a sample size ranging from 102 to 9400. Nine of the studies were conducted in the USA and six in Europe. For case ascertainment, 11 studies had a prospective study design, and 5 had a retrospective study design. Four types of surgical procedures including general surgery, hip surgery, cardiac surgery and lower extremity amputations were involved. Seven studies investigated patients more than 70 years in age, and 9 less than 70 years in age. Ten studies applied univariate analysis and 6 studies used multivariate analysis as statistical method. The NOS scores for the assessment of methodological quality for cohort studies ranged from 5 to 8, with scores ≥ 6 in 14 studies and scores < 6 in 2 studies. The NOS score for the included studies were summarized in Table 3.

**Table 1:** Characteristics of the included studies on the risk of bedsore in diabetic patients undergoing surgery

Author	Year	Region	Study design	Inclusion period	Opertion location	No.of participants	Sex	Mean/median age(ys)	Body mass index	Treatment regimen	Analysis	Follow up period (months)	Adjustment variables
Zambonato	2013	Brasil	Retrospective cohort study	2005-2006	All	1503	F: 52.7%	Mean 55.5±16	NR	NR	Univariate	NR	NR
Ekström	2013	Sweden	Prospective cohort study	NR	Hip fracture	2133	F: 69.50%(DM);72.90% (non DM)	DM:81.6±8.5; non DM:81.2±10.8	NR	NR	Multivariate	2 years	Age, gender, ASA classification,surgical method, type of diabetes and fracture
Tschannen	2012	USA	Prospective cohort study	2007-2009	All	3225	F: 40.8%	Mean: 58.9±16.0	28.7±7.1	NR	Multivariate	NR	Age, sex, BMI, Braden score at admission, risk of mortality, use of vasopressors, number of surgeries, and total operating room time.
Bulfone	2012	Italy	Prospective cohort study	2009	All	102	F:38.2%	Mean:62.3±14.3	26.7±5.4	NR	Univariate	NR	NR
Norris	2011	UK	Prospective cohort study	1989-2008	Hip fracture	5966	Non DM:78.2%; Diabetics on insulin:75.8%;Diabetics diet/tablet controlled (DDTC):78.6%	Non DM:80.0; DM on insulin:75.0;DM diet/tablet controlled (DDTC):79.8	NR	NR	Univariate	1 year	NR
Norris	2011	UK	Prospective cohort study	1989-2008	Hip fracture	102	Non DM:78.2%; Diabetics on insulin:75.8%;Diabetics diet/tablet controlled (DDTC):78.6%	Non DM:80.0; Diabetics on insulin:75.0;Diabetics diet/tablet controlled (DDTC):79.8	NR	NR	Univariate	1 year	NR
Slowikowski	2010	USA	Prospective cohort study	2005-2008	All	102	F:43.6%	Mean:58.3±19.3	28.6±9.0	NR	Multivariate	NR	Not repositioned, age 70, edema, ventilator support, orthotics, and hemodialysis or continuous renal replacement therapy,Braden Scale score
Aragón-Sánchez	2010	Spain	Retrospective cohort study	1998-2008	Lower Extremity Amputations	102	F:63.6%;Non DM: 64.5%;DM: 63%	Median:74	NR	NR	Univariate	NR	Age, heart disease, dislipidemia, high blood pressure, previous amputation, time from the previous major Amputation
Haleem	2008	UK	Prospective cohort study	1989-2006	Hip fractures	102	PS F:75.3% ;NonPS F:78.6%	PS mean:82.1;Non PS mean:76.6	NR	NR	Univariate	NR	NR
Frankel	2007	USA	Retrospective cohort study	NR	All	102	PS F:52.0%; Non PS F: 43.8%	PS mean:67.1; Non PS mean:57.4	NR	NR	Multivariate	NR	Age, gender, Apache II score, serum creatinine, blood urea nitrogen, vascular service admission, presence of a spinal cord injury, and vasopressor requirement during the ICU admission
Pokorny	2003	USA	Prospective cohort study	1997-1998	Cardiovascular surgery	102	PS F:58.0%; Non PS F: 35%	PS mean: 72±5.9; Non PS mean:63±11.0	NR	NR	Univariate	NR	NR
Baumgarten	2003	USA	Retrospective cohort study	1983-1993	hip fracture	9400	F:78.7%	Ages 80 years or more:55.6%	NR	NR	Multivariate	NR	Age, female, ADL score, confused, cachexia or malnutrition,Charlson Comorbidity Index
Spittle	2001	UK	retrospective survey	1995-1998	lower limb amputations	122	DM F:34.8% Non DM F:35%	PS DM mean: 73.5±8.0;Non PS DM mean:68.5±10.2; PS non DM mean:77.3t6.1; Non PS Non DM mean:71.2t10.8	NR	NR	Univariate	NR	NR
Schultz	1999	USA	Prospective cohort study	NR	All	413	Non PS F:34.6%; PS F:39.3%	Non PS:64.4+12.5; PS:70.7+10.1	Non PS:27.39+4.81; PS:25.76+4.26	NR	Multivariate	NR	Age,admission Branden Scale score,body mass, Mattress overlay, newer bed
Stordeur	1998	Belgique	Prospective cohort study	1995	Cardiovascular surgery	163	F:27.6%	64.5+11.3	Non PS:25.6+3.5 ;PS:26.3+4.7	NR	Univariate	NR	NR
Lewicki	1997	USA	Prospective study	NR	Cardiac surgery	337	F:75.4%	Mean:62+11.59	NR	NR	Univariate	NR	NR
Papantonio	1994	USA	Prospective study	NR	Cardiac surgery	136	F:34%	61.9	Non PS:26.6+6.5; PS: 26.5+5.2	NR	Univariate	NR	NR

Abbreviations: ASA, American Society of Anesthesiologists; BMI, body mass index; DM, diabetic mellitus; F, female; M, male; NR, not reported; PS, pressure sores.

**Table 2:** Subgroup analyses of the associations between diabetes and the risk of bedsore in patients undergoing surgery

Variables	RR	95% CI	Degree of heterogeneity (I^2^ statistics; %)	*P*	No. of included Studies	*P*^a^
Total	1.77	1.45 to 2.16	62.7	< 0.001	16	
Study quality						0.009
Score ≥ 6	1.72	1.40 to 2.10	58.1	0.002	14	
< 6	2.07	1.04 to 4.14	65.4	0.089	2	
Surgery type						0.238
General surgery	1.71	1.40 to 2.09	0	0.496	6	
Hip surgery	1.78	1.14 to 2.78	88.4	< 0.001	4	
Cardiac surgery	1.98	1.41 to 2.79	0	0.859	4	
LEAs	1.44	0.93 to 2.24	0	0.414	2	
Study design						0.017
Prospective	1.96	1.52 to 2.52	68.3	< 0.001	11	
Retrospective	1.31	1.07 to 1.59	2.9	0.398	5	
Sample size						0.019
≥ 1000	1.66	1.21 to 2.29	82.6	< 0.001	6	
< 1000	1.93	1.57 to 2.38	0	0.856	10	
Research region						0.523
Europe	1.94	1.26 to 2.99	80.8	< 0.001	6	
USA	1.62	1.33 to 1.97	30.8	0.162	9	
Inclusion period						0.089
Before year 2000	1.38	1.09 to 1.76	16.8	0.308	4	
After year 2000	1.61	1.30 to 2.00	0	0.575	4	
Age						0.078
≥ 70	1.66	1.19 to 2.32	78.3	< 0.001	7	
< 70	1.77	1.48 to 2.11	0	0.695	9	
Analysis method						< 0.001
Univariate	2.08	1.73 to 2.50	10.6	0.342	10	
Multivariate	1.44	1.11 to 1.88	64.8	0.014	6	

Abbreviations: CI, confidence interval; LEA, Lower Extremity Amputations; RR, relative risk.

*P*^a^: *P* values from the test of homogeneity between strata.

**Table 3:** Quality assessment of the included studies

			Selection				Comparability	Outcome			
Study ID			Represent ativeness of the exposed cohort	Selection of the non exposed cohort	Ascertain ment of exposure	Demonstration that outcome of interest was not present at start of study	Comparability of cohorts on the basis of the design or analysis	Assessment of outcome	Was follow-up long enough for outcomes to occur	Adequacy of follow up of cohorts	Quality score
1	Zambonato	2013		★		★	★		★	★	5
2	Ekström	2013		★	★	★	★★	★	★	★	8
3	Tschannen	2012		★	★	★	★★	★	★	★	8
4	Bulfone	2012		★		★	★	★	★	★	6
5	Norris-DOI	2011		★	★	★	★		★	★	6
6	Slowikowski	2010		★	★	★	★★	★	★	★	8
7	Aragón-Sánchez	2010		★	★	★	★		★	★	6
8	Haleem	2008		★			★	★	★	★	5
9	Frankel	2007		★		★	★★	★	★	★	7
10	Pokorny	2003		★		★	★	★	★	★	6
11	Baumgarten	2003		★	★	★	★★	★	★	★	8
12	Spittle	2001		★		★	★	★	★	★	6
13	Schultz	1999		★	★	★	★★	★	★	★	8
14	Stordeur	1998		★		★	★	★	★	★	6
15	Lewicki-preoperative	1997		★		★	★	★	★	★	6
16	Papantonio	1994		★	★	★	★	★	★	★	7

Newcastle-Ottawa Scale for assessing the quality of studies in meta-analysis^⋆^.

Note: A study can be awarded a maximum of one star for each numbered item within the Selection and Outcome categories. A maximum of two stars can be given for Comparability.

### Relationship between diabetes and risk of bedsore

**Figure 2:**
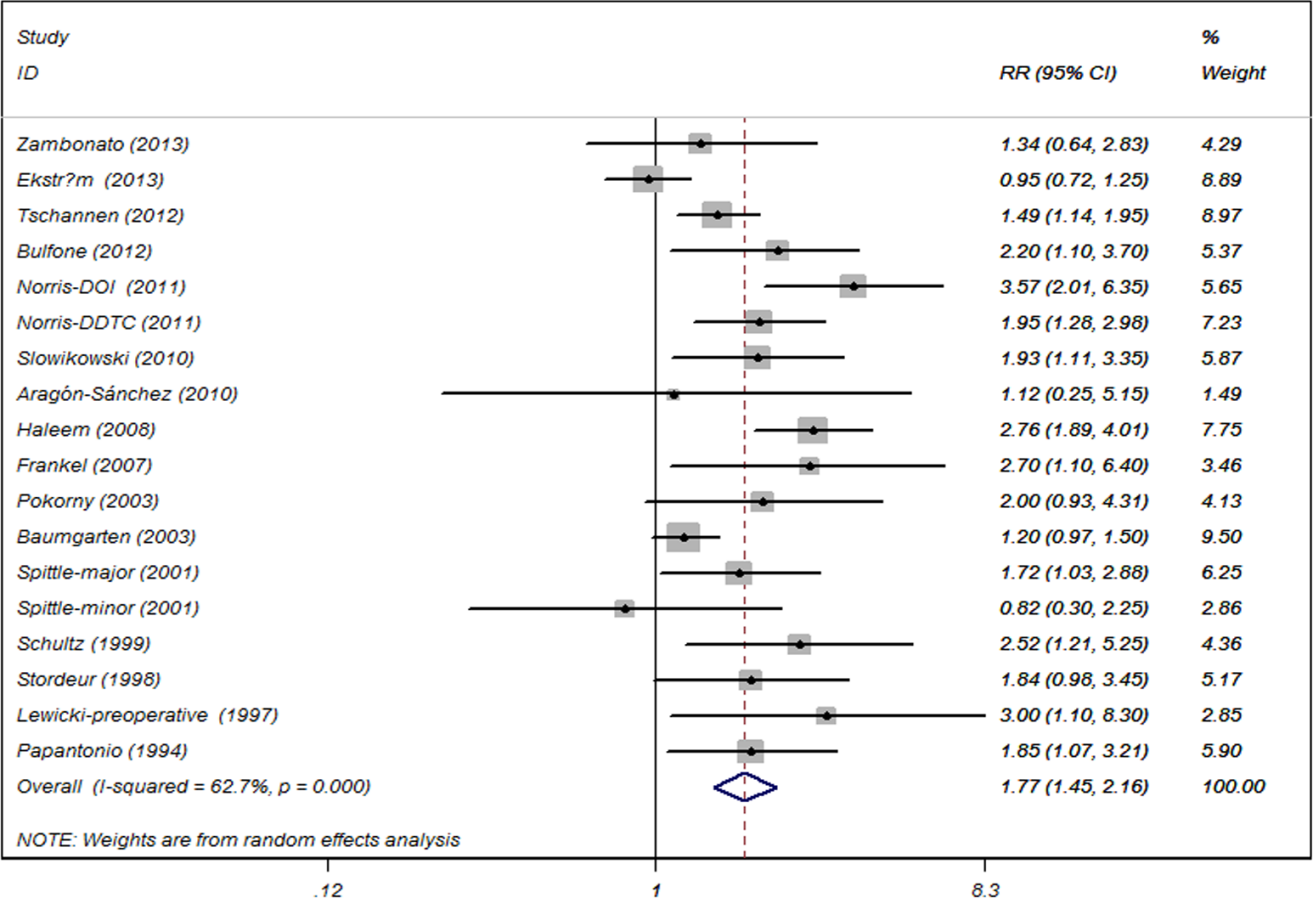
Association between diabetes and the risk of bedsore in patients undergoing surgery

Sixteeen cohort studies investigating the relationship between diabetes and risk of bedsore in surgical patients were included in our meta-analysis. The pooled RR was 1.77 (95% CI, 1.45 to 2.16) and there was statistical inter-study heterogeneity (I^2^ = 62.7%; *P <* 0.001) (Figure 2).

### Methodological quality of the studies

Table 2 presented the summary RRs for bedsore risk and diabetes from 14 high quality studies (≥ 6) and two low quality studies (< 6). In terms of methodological quality of studies, the summary RRs of bedsore risk were 1.72 (95% CI 1.40 to 2.10) in high quality studies and 2.07 (95% CI 1.04 to 4.14) in low quality studies, respectively, in comparison between surgical patients with diabetes and without diabetes. There was statistically significant difference for inter-study heterogeneity (*P* = 0.009).

### Type of surgery

Four types of sugery were involved in the studies, with 6 of general surgery, 4 of hip surgery, 4 of cardiac surgery and 2 of lower extremity amputations, respectively. The summary RRs estimated for bedsore incidence were 1.71 (95% CI 1.40 to 2.09) for general surgery, 1.78 (95% CI 1.14 to 2.78 ) for hip surgery, 1.98 (95% CI 1.41 to 2.79) for cardiac surgery and 1.44 (95% CI 0.93 to 2.24) for lower extremity amputations, respectively. No statistically significant difference for inter-study heterogeneity (*P* = 0.238) was noted.

### Study design

As demonstrated in Table 2, the pooled RRs evaluated for bedsore risk were 1.96 (95% CI 1.52 to 2.52) for prospective studies and 1.31 (95% CI 1.07 to 1.59) for retrospective studies, respectively, with no significant difference for inter-study heterogeneity (*P* = 0.017).

### Sample size

The summarised RRs for bedsore risk stratified by sample size were 1.66 (95% CI 1.21 to 2.29) for studies with large sample size (≥ 1000) and 1.93 (95% CI 1.57 to 2.38) for studies with small sample size (< 1000). We found statistically significant difference for inter-study heterogeneity (*P* = 0.019).

### Research region

Six and 9 studies were conducted in Europe and USA, respectively. The summary RRs for bedsore risk were 1.94 (95% CI 1.26 to 2.99) for studies conducted in Europe and 1.62 (95% CI 1.33 to 1.97) for studies conducted in USA. No statistically significant difference for inter-study heterogeneity was found (*P* = 0.523).

### Inclusion period

Four studies included participants before year 2000 and the pooled RR was 1.38 (95% CI 1.09 to 1.76); and 4 included participants after year 2000 with the pooled RR of 1.61 (95% CI 1.30 to 2.00). We did not find statistically significant difference for inter-study heterogeneity (*P* = 0.089).

### Age

The summarised RRs for bedsore risk stratified by patient age were 1.66 (95% CI 1.19 to 2.32) for studies with patients having older age (≥ 70 years) and 1.77 (95% CI 1.48 to 2.11) for studies with patients having less older age (< 70). There was no statistically significant difference for inter-study heterogeneity (*P* = 0.078).

### Statistical analysis method

Ten studies applied univariate analysis to analyze the risk estimates and 6 studies applied multivariate analysis. The results showed that the pooled RRs for bedsore risk were 2.08 (95% CI 1.73 to 2.50) for studies with univariate analysis and 1.44 (95% CI 1.11 to 1.88) for studies with multivariate analysis. Statistically significant difference for inter-study heterogeneity (*P <* 0.001) was found.

### Publication bias and sensitivity analyses

The shape of the funnel plot for the studies on the diabetes and bedsore risk seemed asymmetrical. In addition, Egger's adjusted rank correlation test showed potential evidence of publication bias (*P* = 0.002). We further test whether publication bias significantly influenced the pooled risk estimates by using trim and filled method and the adjusted RR indicated the same trend with the result of the primary analysis (RR 1.17 95% CI 1.02 to 1.36). A sensitivity analysis was carried out by excluding one study at each time and then recalculating the *pooled* RRs for the remaining ones to test the effect of each study on the overall estimates. We did not find the alteration of in the direction of the estimate when any one of the included study was excluded. This analysis confirmed the robustness of the positive association between diabetes and bedsore risk in surgical patients.

## DISCUSSION

Our systematic review and meta-analysis summarizing the results of 16 observational studies, which comprised a total of 24,112 participants on the association between diabetes and risk of bedsore support the evidence that the risk of developing bedsore among surgical patients exposed to diabetes was 1.77 times that of the non-exposed patients. Analyses stratified by surgical site suggest a greater risk increase for cardiac surgery than for other three investigated surgeries (general surgery, hip surgery or lower extremity amputations), though no statistical significance is found among different surgery types in this meta-analysis.

This updated meta-analysis further confirms and extends the preliminary findings of the two previous published meta-analyses [24, 25]. The first one performed by Liu et al. [25], reported a 115% (OR, 2.15; 95% CI 1.62 to 2.84) higher risk of surgery-related bedsore in diabetic patients compared with that of in non-diabetic patients. The other one conducted by Kang et al. found similar result (surgery related bedsore risk: diabates vs. non-diabetes OR, 1.74; 95% CI 1.40 to 2.15) [24]. Our findings are consistent with the results of previous systematic reviews. We also explored the effect of different surgery types and other potential variables more thoroughly on the combined estimates than the previous ones. Compared with the study by Kang et al., our meta-analysis has added more statistical power to test the surgery type subgroup and examined some other variables which could explain the potential heterogeneity. This study found that diabetic patients having general surgery, hip surgery and cardiac surgery all had significant higher bedsore risk than non-diabetic patients. However, we did not find that association for patients having lower extremity amputations.

Multiple mechanisms can contribute to the deveopment and severity of bedsores, which may result from capillary occlusion by external pressure, leading to the shut off of blood supply, cell death, necrosis removal and ulceration. The severity of the bedsore is determined by the length of time pressure is applied to the local region. Moreover, for a patient receiving surgery, the incidence rate of bedsores is mainly determined by the duration and intensity of the shearing force given upon the tissue during surgery. For the impact of different surgery types on the risk of bedsore development, we noted that patients with cardiact surgery had the higher risk (RR 1.98, 95% CI 1.41 to 2.79) than patients with general surgery or hip surgery, while patients with lower extremity amputations had the lowest risk (RR 1.44, 95% CI 0.93 to 2.24). We propose that the trauma severity of surgery to the body could be a major influential factor determining the risk of developing bedsore.

We noted moderate inter-study heterogeneity in our meta-analysis (I^2^ = 62.7%, *P*_heterogeneity_ < 0.001). Sensitivity analyses indicated that exclusion of any one of the study did not significantly alter the summary estimate. The trim-and-fill model and multiple subgroup analyses stratified by some main clinical variables were in agreement with the initial findings, indicating that the result of this meta-analysis was robust and not affected by publication bias. Nevertheless, we should interpret the results with caution due to the common occurance of publication bias [30] and statistical tests to detect publication bias are incomplete.

Despite the previous published studies investigating the association between diabetes and risk of surgery-related bedsore, the statistical power was quite limited for the small sample sizes of these studies (ranging from 67 to 616). To the best of our knowledge, our study is the most comprehensive one with the largest sample size to evaluate this association. Furthermore, exhaustive search strategies were developed to garantee the inclusion of almost all of the eligible studies, generating 16 studies and data from 24,112 individuals. Such a large sample size could provide us a precise and important risk estimates. Moreover, based on the subgroup analyses, our study also showed that

bedsore risk increased among different types of surgery although statistical significance was not noted for lower extremity amputations probably due to limited sample size. Lastly, consistent and stable sensitivity analyses and result of trim and filled method made the results more strengthened.

Several limitations in our study should be acknowledged. First, variations of treatment or nursing procedures for different types of surgery, may result in variations in risk estimates. Secondly, in order to assess the effect of different blood glucose levels or patient body mass index on the different risk of bedsore, related subgroup analyses should ideally be performed. However, due to the nature of study-level data instead of patient-level data, the available data did not allow us to conduct such assessment. Thirdly, 10 of 16 studies used univariate analysis instead of multivariate analysis to obtain the risk estimates as they did not adjust for some potential influential confounders, such as gender, patient age, diabetes duration and type, which could lead to inaccurately generating the pooled estimates. In addition, for the studies using multivariate analysis, the adjustment variables varied considerably. Moreover, the data sources from observational studies restricted the power to fully explore the influence of unmeasured confounding variables and observational studies could not establish a causal relationship between exposure factor of diabetes and risk of bedsore. Finally, some of the study authors could not be contacted for retrieving some necessary data. Despite the limitations of the current study, the major clinical implication lies in that for some types of surgery, clinicians should take more care of patients with diabetes to mininize the development of surgery-related bedsore and improve the quality of patient life during hospitalization.

In conclusion, our systematic review and meta-analysis provide evidence that diabetic patients having surgery could have a higher risk of developing bedsore. This association is almost independent of surgery type and other study characteristics. However, further large-scale prospective studies should be implemented to further test the association.

## MATERIALS AND METHODS

### Search strategy

We systematically searched Pubmed, EMBASE, and the Cochrane Library without language restriction through November 2016 for related peer-reviewed studies that examined an association between diabetes and risk of bedsore in patients undergoing surgery. We performed this systematic review and meta-analysis based on the Preferred Reporting Items for Systematic Reviews and Meta-Analyses (PRISMA) (Supplementary Table S4) [31]. Two authors (M.L. and Q.C.) independently conducted the literature search using the terms: (surgery OR surgical OR operation OR operative) AND (diabetes mellitus OR diabetes) AND (pressure sore* OR pressure ulcer* OR bedsore* OR decubitus). Manual searches of reference lists of relevant studies obtained from the initial searches were also conducted for some missing citations. Detailed search strategies of each database are provided in Supplementary Appendix.

### Study selection

Two reviewers (M.L. and Q.C.) independently assessed all records through reading the titles and/or abstracts for potentially eligible studies. In case there were different opinions, a senior reviewer (L.L ) would join to discuss and resolve the disagreement. We included studies in this meta-analysis if they satisfied the following criteria: (i) observational studies including cohort or case–control studies; (ii) investigating diabetes and risk of bedsore in patients undergoing surgery; (iii) providing odds ratios (ORs)/relative risks (RRs) along with 95% confidence intervals (95% CIs) or sufficient information to calculate them, for bedsore risk stratified by diabetes in patients having operation. We included patients with history or diagnosis of diabetes, irrespective of diabetes type (1 or 2), disease severity, duration or anti-diabetic drug use due to unavailability of those data.

### Data extraction and quality assessment

Data were extracted independently according to a predesigned form by two reviewers (Y.Z. and L.H.) and the results were crosschecked. A third reviewer(L.L.) would reevaluated the extracted data if any disagreements occurred. The following data were extracted from each study: first author, publication year, study region, study design, inclusion period, opertion site, number of participants, sex, mean/median age, body mass index, treatment regimen, analysis method, follow up period, adjustment variables, and risk estimates for association between bedsore risk and diabetes in patients having operation.

Two reviewers (M.L. and L.H.) independently assessed the methodological quality of each included study using Newcastle-Ottawa quality assessment

scale (NOS) for cohort studies, which included 3 domains (4 points for selection, 2 points for comparability and 3 points for exposure/outcome) totaling 9 points (Table 3). We categoried score less than 6 as low quality and score of 6 or more than 6 as high quality. Discrepancies were resolved by consensus with a senior reviewer (L.L.).

### Statistical analyses

We quantified the relationship between diabetes and risk of bedsore using an inverse variance method using DerSimonian and Laird random-effects models [32]. All statistical analyses were carried out with Stata Statistical Software (version 12.0; StataCorp LP, College Station, TX, USA) by two reviewers (M.L. and L.H.). Between-study heterogeneity was assessed using the chi-square statistic and quantified by I^2^, with an I^2^ statistic more than 50% defining significant heterogeneity [33, 34]. We further investigated potential sources of between-study heterogeneity by subgroup analyses based on some baseline variables (study quality, surgery type, study design, sample size, research region, inclusion period, patient age and analysis method). Egger's regression model was quantified to assess publication bias [35]. If publication bias existed, we used the trim-and-fill method to adjust the pooled estimates of the potential unpublished studies in the meta-analysis, which were compared with the original pooled RRs [36]. Sensitivity analysis was also conducted to investigate the influence of each study on the separate analyses of cohort studies [26]. All statistical analyses were two-sided with a *P* value less than 0.05 indicating significant difference.

## SUPPLEMENTARY MATERIALS FIGURES AND TABLES




