# Supplementary material for: Impact of diabetes on the risk of bedsore in patients undergoing surgery: an updated quantitative analysis of cohort studies
Source: Oncotarget. 2016 Dec 27;8(9):14516–24. doi: 10.18632/oncotarget.14312 (PMC5362422; doi:10.18632/oncotarget.14312)
Supplement: Supplementary file 1 [file oncotarget-08-14516-s001.pdf]

Impact of diabetes on the risk of bedsore in patients undergoing surgery: an updated quantitative analysis of cohort studies

Supplementary Materials

Supplementary Appendix:

Search strategy

Search included: Pubmed, Embase, the Cochrane Library Central Register of Controlled Trials: till November 10, 2016.

Supplementary Table SA1: Search strategy for pubmed (publication date to 2016/11/10)

1. "Surgical Procedures, Operative"[Mesh]
2. "General Surgery"[Mesh]
3. 1 OR 2
4. (Surgery OR Surgical OR Surgeries OR Operation OR Operative)[Title/Abstract]
5. 3 OR 4
6. "Diabetes Mellitus"[Mesh]
7. (Diabetes Mellitus OR Diabetes)[Title/Abstract]
8. 6 OR 7
9. "Pressure Ulcer"[Mesh]
10.(pressure sore* OR pressure ulcer* OR Bedsore* OR decubitus)[Title/Abstract]
11. 9 OR 10
12. 5 AND 8 AND 11

Supplementary Table SA2: Search strategy for embase (publication date to 2016/11/10)

1. 'surgery'/exp
2. (Surgery OR Surgical OR Surgeries OR Operation OR Operative):ab,ti
3. #1 OR #2
4. 'diabetes mellitus'/exp
5. ('Diabetes Mellitus' OR Diabetes):ab,ti
6. #4 OR #5
7. 'decubitus'/exp
8. ('pressure ulcer' or 'pressure ulcers' or Bedsore* or 'Pressure Sore' or 'Pressure Sores' or 'Bed Sore' or 'Bed Sores' or decubitus or 'decubitus ulcer' or 'decubitus ulcers'):ab,ti
9. #6 OR #7
10. #3 AND #6 AND #9

Supplementary Table SA3: Search strategy for the cochrane library central register of controlled trials (publication date to 2016/11/10)

1. MeSH descriptor: [General Surgery] explode all trees
2. Surgery or Surgical or Surgeries or Operation or Operative:ti,ab,kw
3. #1 or #2
4. MeSH descriptor: [Diabetes Mellitus] explode all trees
5.'Diabetes Mellitus' or Diabetes:ti,ab,kw
6. #4 or #5
7. MeSH descriptor: [Pressure Ulcer] explode all trees
8. 'pressure ulcer' or 'pressure ulcers' or Bedsore* or 'Pressure Sore' or 'Pressure Sores' or 'Bed Sore' or 'Bed Sores' or decubitus or 'decubitus ulcer' or 'decubitus ulcers':ti,ab,kw
9. #7 or #8
10. #3 and #6 and #9

Supplementary Table S4: PRIMA 2009 Check list. See Supplementary_Table_S4
